# Methylthioadenosine Reprograms Macrophage Activation through Adenosine Receptor Stimulation

**DOI:** 10.1371/journal.pone.0104210

**Published:** 2014-08-12

**Authors:** Peter A. Keyel, Matthew Romero, Wenbo Wu, Daniel H. Kwak, Qin Zhu, Xinyu Liu, Russell D. Salter

**Affiliations:** 1 Department of Immunology, University of Pittsburgh School of Medicine, Pittsburgh, Pennsylvania, United States of America; 2 Department of Biological Sciences, Texas Tech University, Lubbock, Texas, United States of America; 3 Department of Chemistry, University of Pittsburgh, Pittsburgh, Pennsylvania, United States of America; University of Leipzig, Germany

## Abstract

Regulation of inflammation is necessary to balance sufficient pathogen clearance with excessive tissue damage. Central to regulating inflammation is the switch from a pro-inflammatory pathway to an anti-inflammatory pathway. Macrophages are well-positioned to initiate this switch, and as such are the target of multiple therapeutics. One such potential therapeutic is methylthioadenosine (MTA), which inhibits TNFα production following LPS stimulation. We found that MTA could block TNFα production by multiple TLR ligands. Further, it prevented surface expression of CD69 and CD86 and reduced NF-KB signaling. We then determined that the mechanism of this action by MTA is signaling through adenosine A2 receptors. A2 receptors and TLR receptors synergized to promote an anti-inflammatory phenotype, as MTA enhanced LPS tolerance. In contrast, IL-1β production and processing was not affected by MTA exposure. Taken together, these data demonstrate that MTA reprograms TLR activation pathways via adenosine receptors to promote resolution of inflammation.

## Introduction

Inflammation is one of the first lines of defense against pathogens. However, excessive or poorly regulated inflammation can itself lead to pathology. To avoid these problems, the immune system has developed a number of checkpoints that indicate when inflammation needs to be halted and resolution needs to begin. Understanding the molecular determinants and interplay of inflammatory and anti-inflammatory processes will increase our ability to limit or promote inflammation therapeutically. The inflammatory response begins upon recognition of pathogens by cells. Pathogens are recognized by pattern-recognition receptors, such as Toll-like receptors (TLRs) that recognize certain pathogen-associated molecular patterns (PAMPs). TLRs can be categorically divided into those which signal through MyD88 or Trif adaptors. In addition, TLRs differ in whether they encounter their ligands at the cell surface or internally, and whether they can induce secretion of Type I interferons [1]. In all cases, TLRs induce NF-KB signaling, and there is a common set of inflammatory genes upregulated by multiple TLRs, including cytokines like TNFα [1]. Surface expression of proteins, such as the costimulatory protein CD86, and the activation receptor CD69, are also upregulated following TLR ligation [1], [2]. Although TLR signaling is highly complex, one consequence of TLR ligation is activation of adaptor molecules such as Trif or MyD88, which in turn leads to the activation of IRAK4, TRAF6, IRAK1 and other molecules [1]. This activation cascade culminates in propagation of signals to the nucleus, including via NF-KB [1].

TLR signaling must be suppressed or redirected to halt inflammation. Although removal of the TLR ligand is one aspect of terminating inflammation, there are situations where this does not occur, such as during chronic infections. In cell culture systems, macrophages can retain the TLR4 ligand lipopolysaccharide (LPS) for days following treatment [3]. To balance and resolve inflammation, there are a number of receptors that promote anti-inflammatory responses and blunt the TLR-induced responses. One such set of receptors are the adenosine receptors, which are G protein-coupled receptors that respond to adenosine and related analogs. There are four adenosine receptors, A1, A2a, A2b and A3, of which A2 receptors have been best studied in macrophages [4]. Following activation, adenosine receptors promote robust anti-inflammatory responses. Adenosine receptors block pro-inflammatory cytokines including TNFα, MIP1, while inducing anti-inflammatory cytokines like IL-10 [4]–[6]. In macrophages, it is primarily the A2a and to a lesser extent A2b receptors that reduce inflammation [4]. A2 receptors promote alternative activation of macrophages [6]–[8]. Adenosine receptors can interact with TLR signaling to promote resolution of inflammation, including upregulation of VEGF [9]. Both MyD88 and A2a receptor signaling is necessary to promote this switch from an inflammatory to a wound-healing phenotype [8]. Independently of MyD88, A2a receptors can reduce NF-KB signaling via cAMP production following receptor activation [10]. This suggests adenosine receptors may serve as part of the cellular resolution phase. There are several lipid species, termed resolvins and protectins, that exert pro-resolving action [11]. Pro-resolving action is local immunosuppression that allows a return of the locally inflamed tissue to homeostasis. Resolvins and protectins lead to removal of neutrophils and promotes phagocytosis of apoptotic neutrophils by macrophages along with inducing anti-inflammatory phenotypes in macrophages [11]. However, it is likely that compounds other than lipids may also exert pro-resolving action.

One possible pro-resolving compound is 5′-deoxy-5′-(methylthio)adenosine (MTA), which plays a number of roles. MTA can serve as a metabolite that can be converted to methionine and is present at 11 µM in plasma [12]–[14]. MTA enhances cell death following infection with Salmonella, which is attributed to its role as a methionine precursor [13]. MTA also has a well-known role as a methyltransferase inhibitor [15], [16]. In this capacity, MTA can alter histone modifications and influence gene expression [15]. Similarly, MTA can inhibit Stat1 methylation during the interferon response [17]. Finally, MTA has a well-known, but poorly-characterized, ability to block TNFα production following LPS stimulation [15], [18], [19]. MTA has been used as an immunosuppressive drug to block colitis, liver disease and autoimmunity in rodent models [14], [18], [20]. Taken together, MTA has the capacity to serve in many roles, though the mechanism of immunosuppression is not known.

MTA has as its backbone, adenosine. This, along with the finding that MTA can inhibit LPS-induced TNFα production, suggests that MTA may also serve as an adenosine receptor agonist. Here we examined the mechanism by which MTA block TNFα production and macrophage activation. We found that MTA blocked macrophage activation. We found that inhibition of adenosine receptors A2a and A2b were sufficient to relieve this inhibition, indicating that MTA acts through adenosine receptors. Finally, we found that MTA modulates the TLR response by enhancing LPS tolerance without altering IL-1β production, maturation or secretion. Taken all together, we demonstrate a novel mechanism by which MTA modulates inflammation.

## Results

**Figure 1:**
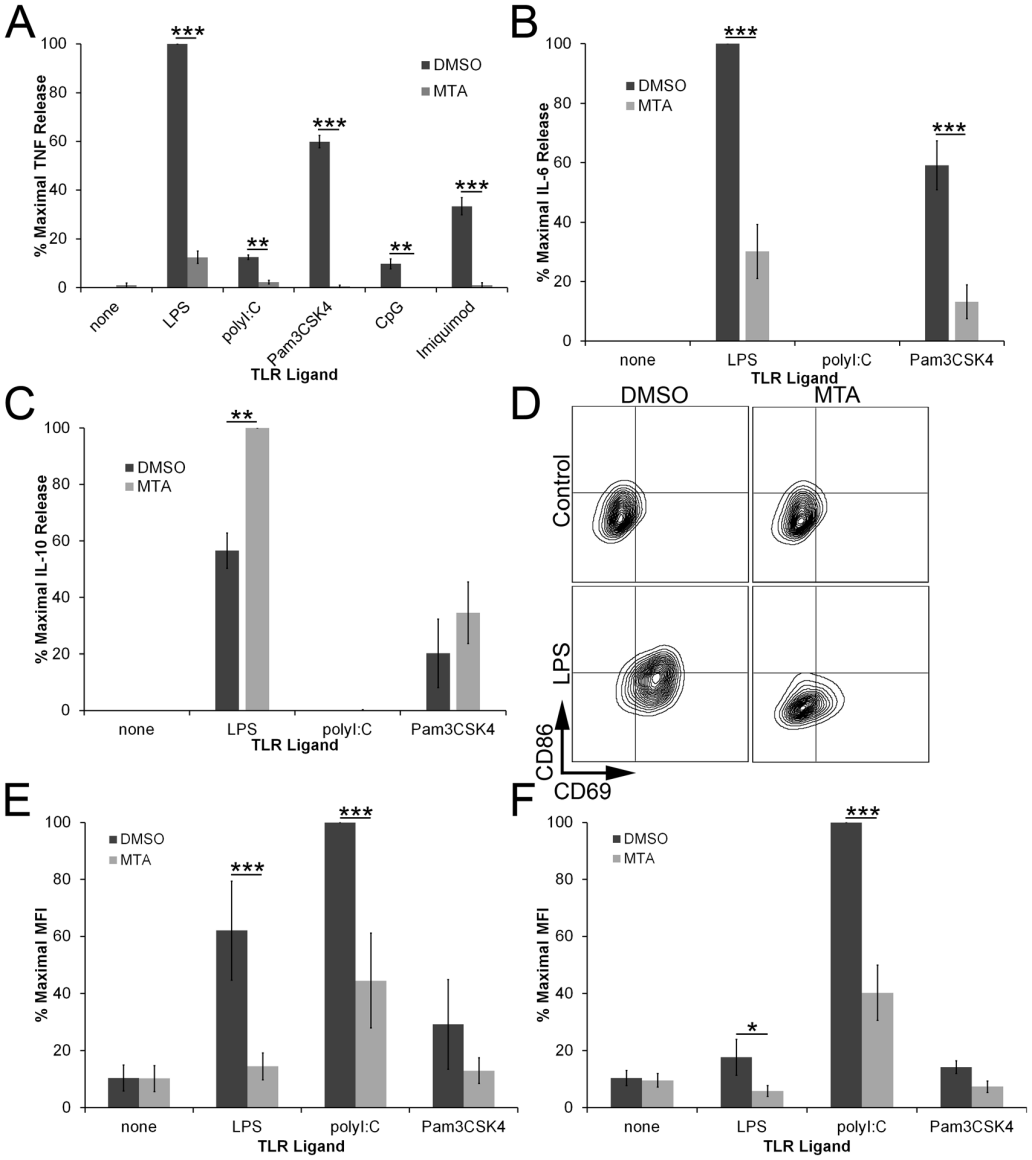
MTA inhibits TLR responses. BMDM were incubated overnight with DMSO or 200 µM MTA in the absence or presence of the following TLR ligands: 10 ng/mL LPS, 10 µg/mL polyI:C, 1 µg/mL Pam3CSK4, 6.67 µM CpG or 2 µg/mL Imiquimod. Supernatants were assayed for TNFα (A), IL-6 (B) or IL-10 (C) production by ELISA while cells were harvested, stained and analyzed for surface CD69 (D,E) or CD86 (D, F) expression by FACS. Graphs represent mean ±sem of 3 (A, E, F), 4 (C) or 5 (B) experiments. * p<0.05, ** p<0.01, *** p<0.001.

Since MTA can inhibit LPS-induced TNFα production in RAW cells and in human PBMC [15], [18], [19], we asked whether MTA could inhibit TNFα production in mouse bone marrow derived macrophages (BMDM) in response to LPS and other TLR ligands. We treated BMDM with TLR2, 3, 4, 7, and 9 agonists overnight in the presence of either DMSO or MTA and measured levels of secreted TNFα (Fig. 1A). We found that MTA inhibited TNFα production due to various TLR ligands (Fig. 1A). We next tested whether MTA altered other inflammatory and regulatory cytokines by testing IL-6 and IL-10 production. We found that TLR4 and TLR2, but not TLR3, stimulation induced IL-6 and IL-10 (Fig. 1B, C). MTA inhibited IL-6 secretion following both TLR2 and TLR4 stimulation (Fig. 1B). As previously reported [19], we found that co-incubation of MTA and LPS increased IL-10 expression (Fig. 1C). However, MTA did not increase IL-10 following TLR2 stimulation (Fig. 1C). To measure the activation status of the BMDM, we used FACS to measure the expression of the activation receptor CD69 [2] and the costimulatory protein CD86 (Fig. 1D–F). We found that MTA inhibited LPS and polyI:C- dependent CD69 and CD86 upregulation (Fig. 1D, E). We found similar results with CD80 (data not shown). MTA did not inhibit Pam3CSK4, CpG or Imiquimod activation because these ligands did not strongly induce either CD69 or CD86 in our assay (Fig. 1D, F and data not shown). Taken together, these data indicate that MTA can specifically suppress the activation of macrophages by ligation of TLRs.

**Figure 2:**
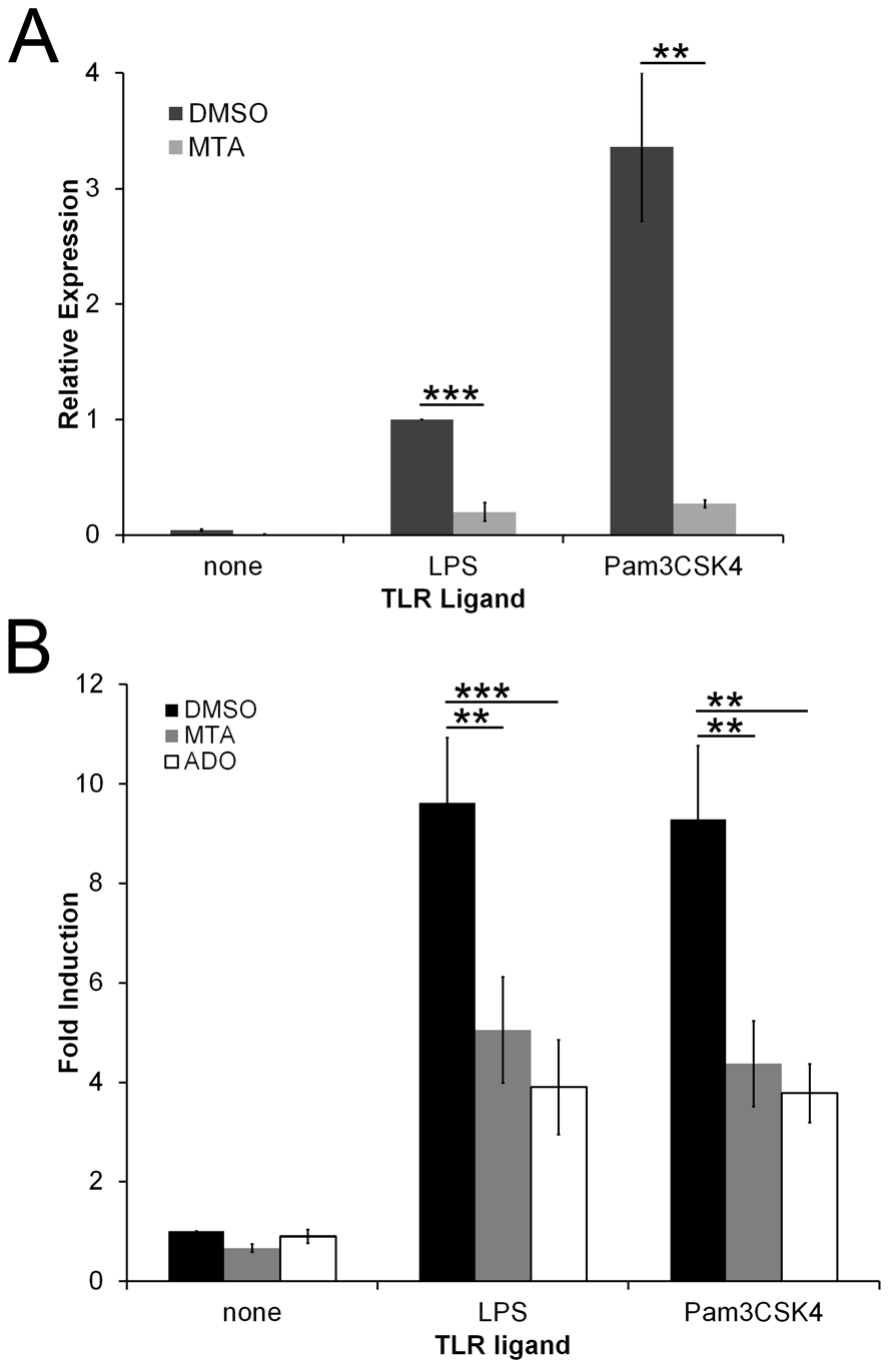
MTA inhibits TLR-induced TNFα mRNA production and NF-KB induction. (A) BMDM were incubated for 4 h with either DMSO or 200 µM MTA in the absence or presence of either 100 EU/mL LPS or 1 µg/mL Pam3CSK4. Total RNA was extracted from cells. TNFα expression relative to β-actin was determined by Δ(ΔCT) method using real-time PCR. (B) RAW NF-KB reporter cells were treated with DMSO, 200 µM MTA or 200 µM adenosine (Ado) in the absence or presence of the indicated TLR ligands for 4 h at 37°C. Supernatants were assayed for luciferase, which was normalized to DMSO-treated cells that received no TLR stimulation. The graphs represent mean ±sem of 4 (A) or 5 (B) experiments. ** p<0.01, *** p<0.001.

To further explore the effect of MTA on macrophage activation, we tested whether MTA altered TNFα mRNA levels. MTA decreased TNFα mRNA 5-fold and 12-fold following LPS and Pam3CSK4 stimulation respectively (Fig. 2A). This is similar to Adenosine (Ado) mediated suppression of TNFα mRNA [21]. This suggested that MTA exerted transcriptional, rather than translational or post-translational, control on cytokine production. We next tested whether MTA altered TLR signaling to the nucleus. We examined NF-KB activation using RAW macrophages that stably express the *Metridia* secretable luciferase under the control of NF-KB response elements [22]. These cells do not respond to TLR3 ligands (data not shown), but produce a 10-fold increase in luciferase activity following LPS or Pam3CSK4 stimulation (Fig. 2B). When TLR-stimulated cells were co-incubated with either Ado or MTA, NF-KB induction was reduced to a 5-fold increase over DMSO-treated cells (Fig. 2B). Importantly, Ado and MTA themselves did not promote detectable NF-KB signaling (Fig. 2B). Since TLRs and A2 receptors synergize to produce an anti-inflammatory phenotype and both require IRAK4 and TRAF6 signaling [8], it is not surprising that Ado and MTA did not completely inhibit NF-KB responses. We conclude that MTA and Ado alter TLR-induced transcriptional responses and NF-KB signaling.

**Figure 3:**
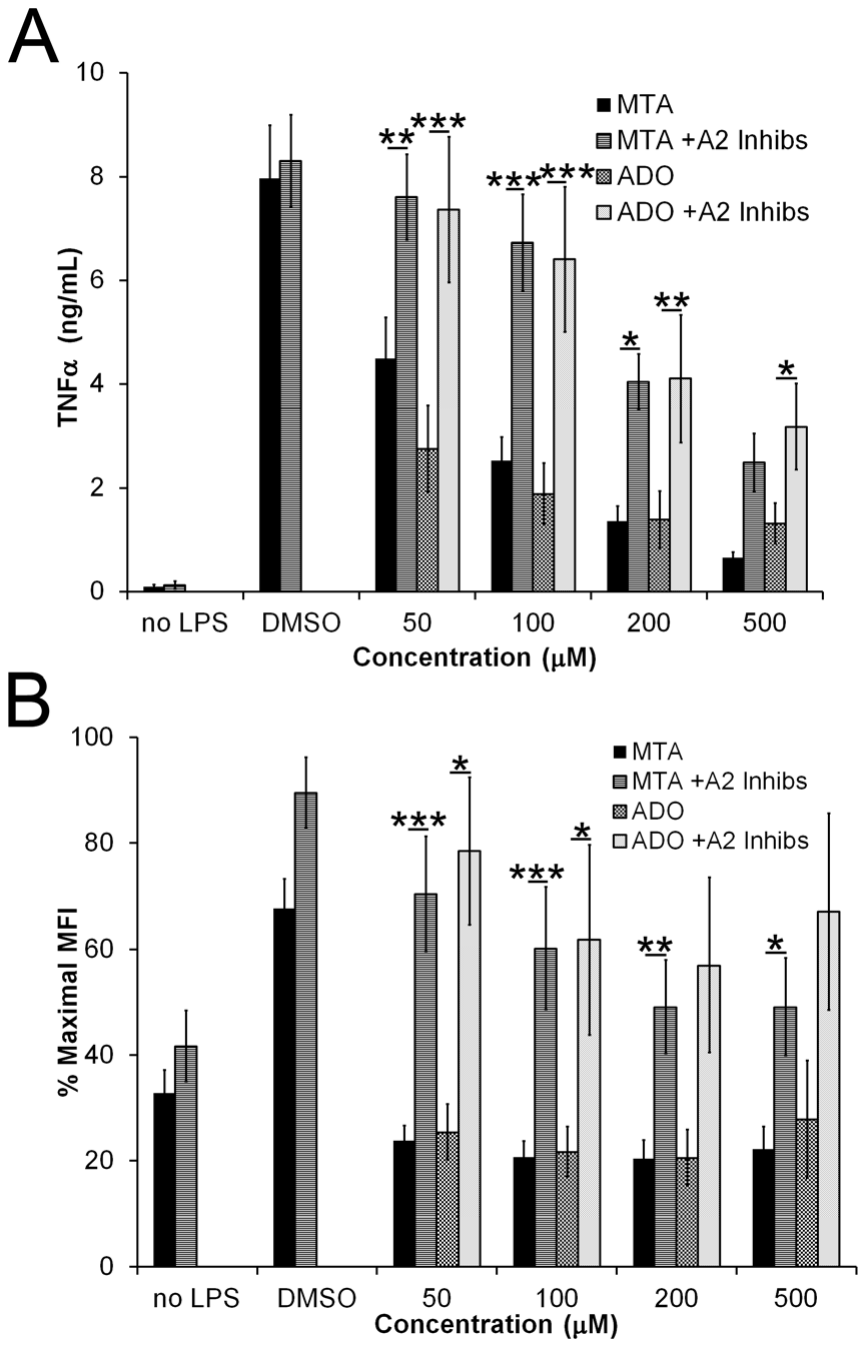
MTA inhibition of TLR ligands acts via Adenosine Receptors. BMDM were incubated overnight with DMSO, the indicated concentrations of MTA or adenosine (Ado), presence or absence of 10 µM SCH442416 and 10 µM PSB1115 and the presence or absence of 10 ng/mL LPS. Supernatants were analyzed for TNFα production by ELISA (A) while cells were harvested, stained and analyzed for surface CD86 (B) expression by FACS. Data represent mean ±sem of at least 3 experiments. * p<0.05, ** p<0.01, *** p<0.001.

We next asked how MTA suppresses TLR-induced signaling. Adenosine receptors are known to alter TLR signaling, including suppressing TNFα [6], [8], [9]. Since MTA is structurally very similar to adenosine, we asked whether adenosine receptors could mediate the inhibitory effect of MTA. A2a and A2b receptors are the primary adenosine receptors responsible for inducing an anti-inflammatory phenotype in macrophages [4]. Although macrophages express A3 adenosine receptor, the A3 inhibitor MRE 3008F20 did not relieve MTA-inhibition of TNFα production (data not shown). We treated BMDM with or without LPS, MTA, adenosine (Ado) in the presence or absence of specific A2a and A2b inhibitors (SCH442416 and PSB1115) overnight at 37°C. We found that BMDM treated with increasing concentrations of either MTA or Ado produced less TNFα than BMDM treated with DMSO alone (Fig. 3A). A2a and A2b inhibitors alone did not alter the amount of TNFα produced by macrophages (Fig. 3A), though these inhibitors alter NF-KB induction and block CD69 upregulation (data not shown). A2 inhibitors did reverse the TNFα inhibition observed with either MTA or Ado, implicating A2 receptors in the mechanism of MTA-mediated inhibition of TLR responses (Fig. 3A). Similarly, we find that A2 receptor inhibitors reverse the effects of MTA or Ado on CD86 expression (Fig. 3B). Taken together, these data indicate that MTA triggers A2 receptors, which are responsible for inhibition of these TLR responses.

**Figure 4:**
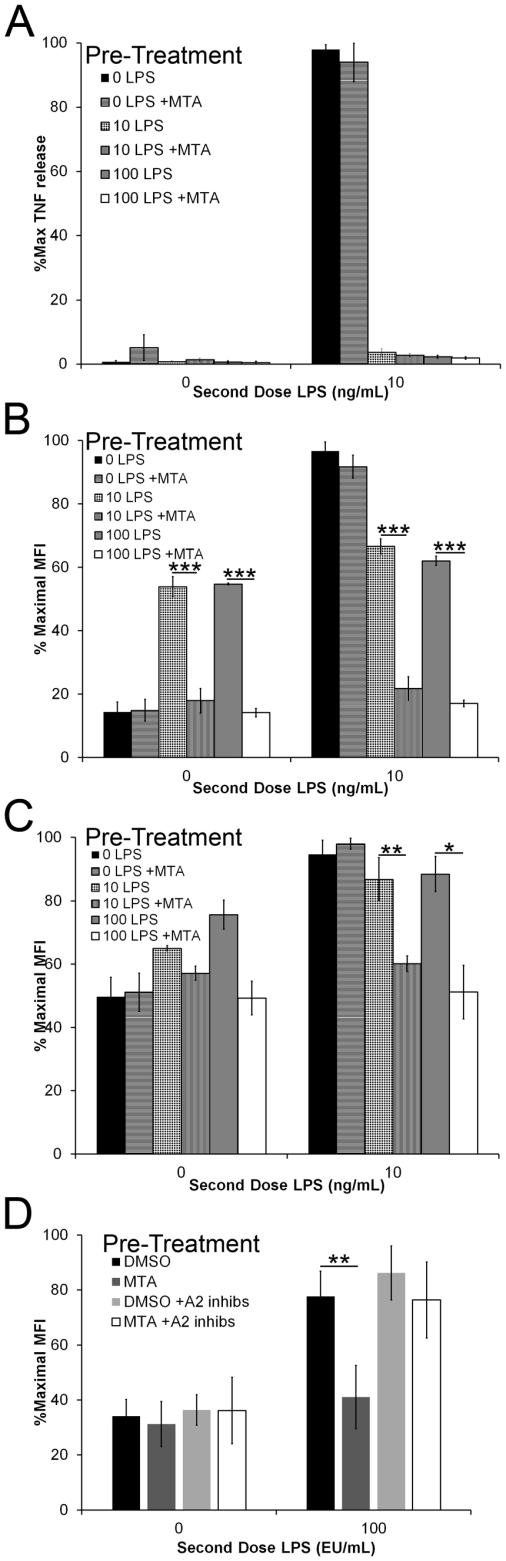
MTA alters LPS tolerance. BMDM were treated overnight with either 0 ng/mL, 10 ng/mL, or 100 ng/mL (A–C) or 100 EU/mL (D) LPS in the presence or absence of 200 µM MTA, and 10 µM SCH442416 with 10 µM PSB1115, washed and rested for 6 h. BMDM were then restimulated with either 0, 10 ng/mL, or 100 EU/mL LPS overnight. Supernatants were collected and analyzed for TNFα (A) by ELISA while cells were harvested, stained and analyzed for surface CD69 (B) or CD86 (C, D) expression by FACS. Data represent mean ±sem of 3 (A) or 4 (B–D) experiments. * p<0.05, ** p<0.01, *** p<0.001.

We next tested whether MTA had a durable effect on TLR induced responses, an important consideration for potential use as a therapy. Prolonged LPS signaling induces a phenomenon termed “LPS tolerance” whereby cells become desensitized to the effects of LPS [23], [24]. We tested whether MTA altered LPS tolerance in BMDM. We first treated BMDM with LPS in the presence or absence of MTA, rested the cells, and then rechallenged them with or without LPS. When cells were pretreated with LPS, they were desensitized to subsequent LPS challenges, failing to produce TNFα (Fig. 4A). Pretreatment with MTA alone did not block subsequent LPS-induced TNFα production (Fig. 4A). MTA also did not block LPS-induced tolerance, as shown by lack of ability to restore TNFα production upon LPS re-exposure (Fig. 4A). We next examined CD69 expression. We found that pretreatment with LPS induced CD69 upregulation which persisted through the second stage of LPS treatment independently of the second LPS treatment (Fig. 4B). Interestingly, MTA did inhibit this CD69 expression, confirming that the MTA was active during this pretreatment (Fig. 4B). However, when LPS was absent during pretreatment, MTA did not affect subsequent CD69 expression, suggesting that MTA must exert its effect on cells at the same time as delivery of a TLR signal (Fig. 4B). We found a distinct effect on CD86 expression. Pretreatment with MTA alone did not alter CD86 levels, and subsequent exposure to LPS-induced CD86 expression normally (Fig. 4C). However, when cells were exposed to MTA in the presence of LPS, subsequent treatment with LPS was unable to up-regulate CD86 (Fig. 4C). We next tested whether MTA altered LPS tolerance in an adenosine receptor dependent fashion. We found that A2 inhibitors prevented MTA-induced suppression of CD86 (Fig. 4D). These findings lead us to hypothesize that synergy between adenosine receptors and TLRs promotes anti-inflammatory signaling. In addition, we conclude that MTA requires concomitant TLR signaling to promote a tolerogenic phenotype.

**Figure 5:**
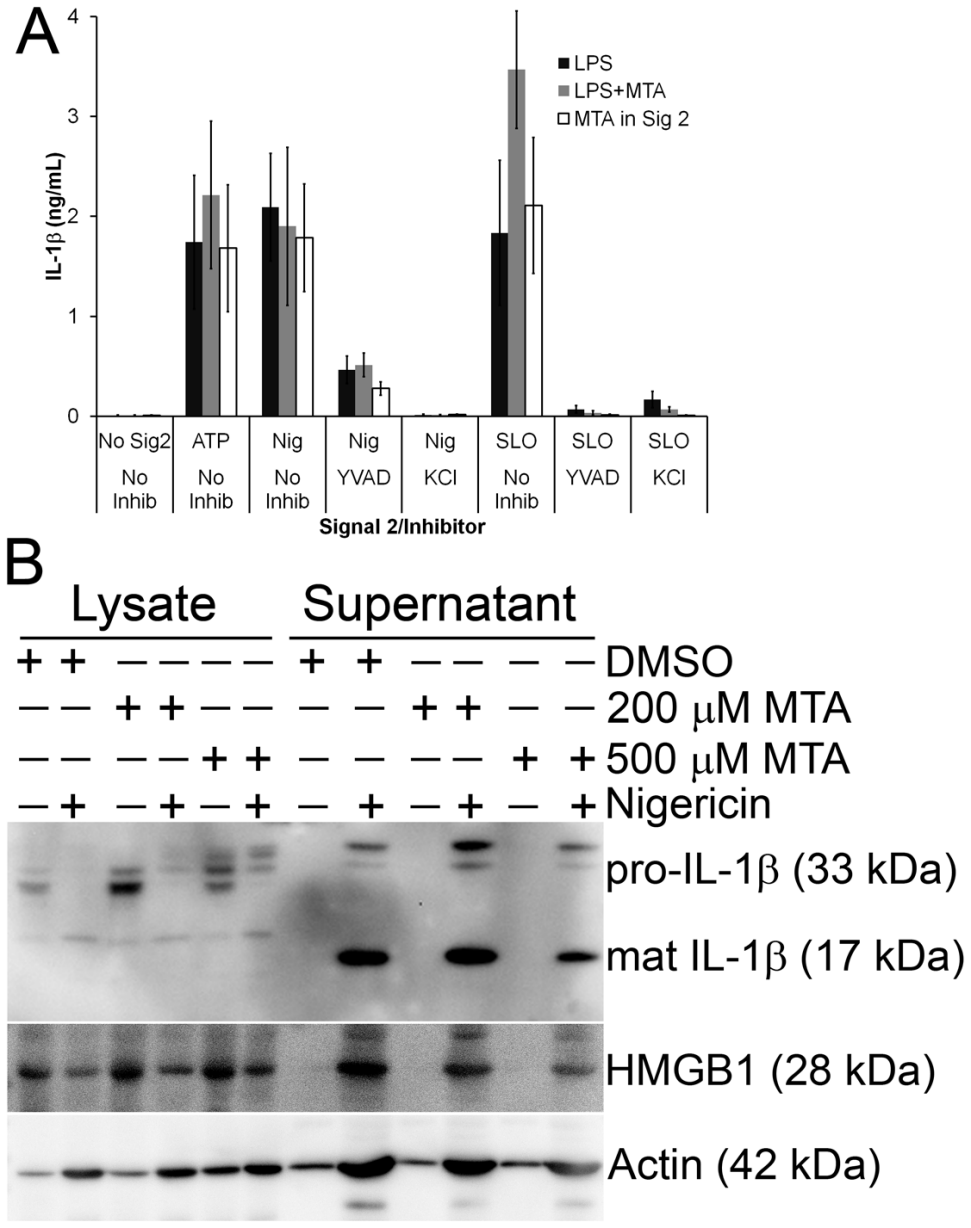
IL-1β secretion is independent of MTA. BMDM were primed for 4 h with 10 ng/mL LPS in the absence or presence of 200 µM or 500 µM MTA, then washed and treated in the presence (MTA in Sig 2) or absence of 200 µM MTA, inflammasome inhibitors YVAD or KCl and NLRP3 agonists 3 mM ATP, 2000 U/mL SLO or 20 µM nigericin for 30 min. Supernatants were collected and analyzed by ELISA (A) or TCA-precipitated, resolved by SDS-PAGE along with cell lysates, and transferred to PVDF (B). Blots were sequentially probed with 3ZD anti-IL-1β mAb, EPR3057 anti-HMGB1 rabbit mAb and anti-actin mAb coupled with relevant HRP-conjugated secondary antibodies. The graph is the mean ± sem of 4 experiments while the blot is a representative blot from 3 independent experiments.

We also tested whether MTA could alter TLR signaling required for IL-1β protein synthesis and secretion. TLR ligation is the first of two steps necessary for IL-1β processing and release [25]. Complete inhibition of TLR signaling should block synthesis of IL-1β protein. In addition, it is possible that MTA could block the second step in IL-1β release, a caspase-1 dependent cleavage dependent on inflammasome activation. To test this, we primed BMDM with LPS in the presence or absence of MTA and then challenged the BMDM with NLRP3 inflammasome agonists ATP, nigericin or streptolysin O (SLO) (Fig. 5A). Surprisingly, we found that inclusion of MTA during the LPS priming stage did not alter IL-1β secretion (Fig. 5A). To ensure that MTA had no effect on the NLRP3 agonists, we LPS-primed BMDM and then challenged them with ATP, nigericin or SLO in the presence or absence of MTA (Fig. 5A). We found no significant differences in the amount of IL-1β secreted by control untreated cells and those exposed to MTA. Treatment with the Caspase-1 inhibitor YVAD or NLRP3 inhibitor KCl abolished IL-1β secretion (Fig. 5A). We next examined IL-1β release by western blot to test whether MTA had any effect on IL-1β processing. We primed BMDM with LPS and either DMSO, 200 µM or 500 µM MTA for 4 hours, washed the cells and challenged them with nigericin for 30 minutes. We found that MTA did not impair the processing and release of IL-1β or the release of the proinflammatory cytokine HMGB1 (Fig. 5B). We conclude that MTA does not alter IL-1β processing or secretion.

## Discussion

Here we have examined the effects of MTA on TLR stimulation. We find that MTA blocks TNFα and IL-6 production and activation receptor expression through adenosine A2 receptors. This tolerogenic effect does not block TLR signaling, so much as redirect it to a different signaling pathway. To this end, we observe that TLR-induced NF-KB signaling is reduced but not ablated, and that LPS tolerance is altered to show a reduction in activation markers. However, we observed no change in IL-1β processing and secretion, indicating that this altered phenotype is the result of a specific cellular program to promote resolution of inflammation.

Although MTA has been described both as a metabolic intermediate and as a methyltransferase inhibitor, we ascribe a novel function to this compound: adenosine receptor activator. MTA may act indirectly by inhibiting adenosine uptake or by directly binding A2 receptors. Either way, we find that MTA exerts its TNF-suppressive effects through A2a and A2b receptors, similarly to the effects Ado can play. This finding adds a caveat on studies that wish to ascribe MTA function entirely to direct methyltransferase activity or role as methionine precursor. A2 receptor signaling will be active under these conditions. The amount of MTA needed to impair TNFα production was 50 µM, and similar to previous results [19], TNFα was blocked in a dose-dependent fashion. Since MTA is typically present at five-fold lower levels in the serum, the body could potentially modulate the inflammatory response by increasing MTA locally or systemically. This would also represent one link between methionine metabolism and innate immunity. Interestingly, all of the MTA treatment effects observed here required concurrent TLR signaling. One possibility for this requirement is feedback between A2a receptor expression and LPS and/or TNFα signaling. In human cell lines, both LPS and TNFα induce A2a receptor mRNA expression, which potentiates the anti-inflammatory effects of the A2a receptor [26]–[28]. Taken all together, these results suggest that MTA may act as a pro-resolving compound.

The anti-inflammatory phenotype described here for MTA is consistent with established adenosine receptor functions. There is a growing body of evidence showing that adenosine receptors induce an anti-inflammatory phenotype [6], [7], [9], [21]. Adenosine receptors can induce T regulatory cell generation [29]. A2 receptors promote the M2 phenotype of macrophages [7], [8], consistent with the suppression of activation markers observed here. A2 receptor signaling interacts with the TLR pathway at the level of IRAK4 and TRAF6, providing a molecular basis for the synergistic anti-inflammatory activity [8]. These data are consistent with our findings that NF-KB induction is reduced, but not eliminated by either MTA or Ado treatment. Adenosine receptors can reduce NF-KB independently of IRAK1 signaling via downstream signaling following cAMP production [10]. Adenosine receptors can also act independently of NF-KB to regulate TNFα levels [21], [30], [31].

We further found that MTA enhanced LPS tolerance. Specifically, MTA prevented the expression of an activation receptor that persisted following initial stimulation and blocked upregulation of the costimulatory molecule CD86. These are new examples of how adenosine receptor signaling can enhance and alter the effects of TLR signaling. It is known that adenosine receptor signaling reprograms the inflammatory response [4], [8]. Generally inflammation moves from a pro-inflammatory response to an anti-inflammatory response. This anti-inflammatory phase is an important part of the resolution phase of the innate immune response [11]. Adenosine receptors may represent part of the switch from pro- to anti-inflammatory response. Enhancing this pathway may lead to novel therapeutics for resolving chronic inflammatory diseases.

Interestingly, MTA failed to inhibit IL-1β. As a switch that also requires TLR signaling, adenosine receptors may represent one of the earliest steps in transitioning from a pro-inflammatory phenotype to an anti-inflammatory one. In this case, certain pro-inflammatory cytokines would be expected to persist during this phase of the switch. At a molecular level, it has recently been shown that IRAK1 is necessary for promoting NLRP3 inflammasome action [32]. Since adenosine receptors require MyD88, IRAK4 and TRAF6 signaling from TLRs for the anti-inflammatory signaling [8], they may not directly influence inflammasome activation. How resolution proceeds after adenosine receptors synergize with TLRs remains to be determined.

## Materials and Methods

### Reagents

All reagents were from Thermo Fisher Scientific (Waltham, MA) unless otherwise noted. Nigericin, adenosine and MTA were from Sigma-Aldrich (St Louis, MO), while SCH442416, PSB1115 and MRE 3008F20 were from Tocris (Minneapolis, MN) and TLR ligands were from Invivogen (San Diego, CA). Anti-HMGB1 rabbit monoclonal antibody (mAb) EPR3507 was from Genetex (Irvine, CA), anti-actin mouse mAb AC-15 was from Sigma-Aldrich, anti-IL-1β mouse mAb 3ZD was from Frederick National Laboratory for Cancer Research (Frederick, MD), IL-1β ELISA antibodies were from eBioscience (San Diego, CA), TNFα, IL-6 and IL-10 ELISA antibodies were from BioLegend (San Diego, CA), FITC-conjugated anti-CD69 and APC-conjugated anti-CD86 mAb were from BD Biosciences (San Jose, CA), and anti-mouse and anti-rabbit antibodies conjugated to HRP were from Jackson Immunoresearch (West Grove, PA). Bone marrow was either a generous gift from Lisa Borghesi or obtained from C57BL/6 mice (Jackson Labs, Bar Harbor, ME) maintained in accordance with the Texas Tech Institutional Animal Care and Use Committee.

### Cell culture

BMDM were isolated and cultured as previously described [33]. All procedures and use of mice in this study were approved by the University of Pittsburgh IACUC committee and conform to national guidelines. Bone marrow was collected from mice after asphyxiation using carbon dioxide by Dr. Peter Keyel, as approved in an IACUC protocol granted to Dr. Lisa Borghesi, University of Pittsburgh. Dr. Keyel is listed in the personnel section of the IACUC protocol and is approved to perform this procedure in our laboratory. Briefly, the bone marrow of C57BL/6 mice was from femora and tibiae, and the cells cultured at 37°C for 7–21 days on bacterial grade plates in 20% fetal calf serum (FCS), 30% L929 cell supernatant, 1× sodium pyruvate, 1× L-glutamine and 1× penicillin/streptomycin in DMEM. The cells were plated into 10% FCS, 1× L-glutamine and 1× penicillin/streptomycin in DMEM (D10) one day prior to experiments. RAW cells stably transfected with the NF-KB reporter have been previously described [22] and were cultured in D10 supplemented with 0.2 mg/mL G418.

### MTA functional assays

BMDM were plated at 2.5×105 cells/well in a 12-well plate one day prior to the experiment. Cells were treated with 200–500 µM MTA or adenosine in the presence or absence of 10 ng/mL LPS, 10 µg/mL polyI:C, 1 µg/mL Pam3CSK4, 6.67 µM CpG or 2 µg/mL Imiquimod along with the presence or absence of 10 µM A2a receptor inhibitor SCH442416 and 10 µM A2b receptor inhibitor PSB1115 overnight at 37°C. Supernatants were collected for evaluation by ELISA, while cells were harvested, stained with FITC-conjugated anti-CD69 and APC-conjugated anti-CD86 and analyzed by flow cytometry. Percent maximal values of median fluorescent intensity was used to normalize results across experiments.

### LPS tolerance

BMDM were plated at 2.5×10^5^ cells/well in a 12-well plate one day prior to the experiment. Cells were treated with or without 200 µM MTA in the presence or absence of 10 ng/mL or 100 ng/mL LPS overnight at 37°C. The following morning, the cells were washed 3× in PBS, rested in D10 for 6 hours at 37°C, washed, and restimulated with 0 or 10 ng/mL LPS overnight at 37°C. Supernatants were collected for evaluation by TNFα ELISA, while cells were harvested, stained with FITC-conjugated anti-CD69 and APC-conjugated anti-CD86 and analyzed by flow cytometry. Alternatively, cells were tolerized with 100 endotoxin units (EU)/mL LPS in the presence or absence of 200 µM MTA, 10 µM SCH442416 and 10 µM PSB1115 prior to restimulation with 100 EU/mL LPS. The change from ng/mL to EU/mL reflects changes to how Invivogen measured LPS quantities during the course of the study.

### Real-time PCR

BMDM were plated at a density of 2×10^6^ in a 6 well plate and stimulated for 4 h with nothing, 100 EU/mL LPS, or 1 µg/mL Pam3CSK4 along with either DMSO or 200 µM MTA. Total RNA was extracted using TRI-Reagent (Life Technologies) and cDNA produced using Superscript III (Life Technologies). TNFα and β-actin was measured on a 7300 Real-time PCR system using SybrGreen (Life Technologies). Primer sequences are available upon request.

### NF-κB induction

RAW NF-κB cells were plated at a density of 2×10^5^ in a 24 well plate and allowed 2 hours at 37°C to settle. The cells were then stimulated with nothing, 10 ng/mL LPS, or 1 µg/mL Pam3CSK4 in the presence or absence of 200 µM MTA or adenosine in RPMI for 4 hours at 37°C. The supernatants were collected and assayed for luciferase activity according to manufacturer's instructions (Clontech, Mountain View, CA). Values were normalized to untreated cells to give fold-induction.

### IL-1βassays

Assays to determine IL-1β production were performed as previously described with slight modifications [33]. 10^5^ (ELISA) or 10^6^ (blot) BMDM were primed with 10 ng/mL LPS in the absence or presence of 200–500 µM MTA for 4 hours at 37°C. BMDM were washed and treated with 20 µM nigericin, 3 mM ATP or 500 U/mL SLO in the presence or absence of 200 µM MTA, 100 µM YVAD or 50 mM KCl for 30 min at 37°C. For ELISA, supernatants were collected and analyzed as previously described [33]. For western blots, samples were prepared and resolved by SDS-PAGE as previously described [33]. PVDF membranes were probed with anti-IL-1β 3ZD mAb or anti-HMGB1 mAb, anti-mouse or anti-rabbit-HRP secondary antibody, and visualized with enhanced chemiluminescent reagent (Santa Cruz Biotechnologies, Santa Cruz, CA). Blots were stripped and reprobed with AC-15 anti-actin mAb and anti-mouse-HRP secondary antibody.

### Statistics

Two-way ANOVA followed by Bonferroni post-testing was performed using Prism (Graphpad, La Jolla, CA).
